# *In Vitro* Activity of Lactobacilli with Probiotic Potential Isolated from Cocoa Fermentation against* Gardnerella vaginalis*

**DOI:** 10.1155/2017/3264194

**Published:** 2017-10-31

**Authors:** Wallace Felipe Blohem Pessoa, Ana Clara Correia Melgaço, Milena Evangelista de Almeida, Louise Pereira Ramos, Rachel Passos Rezende, Carla Cristina Romano

**Affiliations:** ^1^Departamento de Ciências Biológicas, Laboratório de Imunologia, Centro de Biotecnologia e Genética, Universidade Estadual de Santa Cruz (UESC), Campus Soane Nazaré de Andrade, Salobrinho, Rodovia Jorge Amado, Km 16, 45662-900 Ilhéus, BA, Brazil; ^2^Departamento de Ciências Biológicas, Laboratório de Biotecnologia Microbiana, Centro de Biotecnologia e Genética, Universidade Estadual de Santa Cruz (UESC), Campus Soane Nazaré de Andrade, Salobrinho, Rodovia Jorge Amado, Km 16, 45662-900 Ilhéus, BA, Brazil

## Abstract

Study of the probiotic potential of microorganisms isolated from fermented foods has been increasing, especially studies related to lactobacilli. In intestinal models, lactobacilli have demonstrated beneficial properties, such as anti-inflammatory activity and increased antibody production, but the molecular mechanisms involving probiotic and antagonistic action as well as their effect on human vaginal cells have not yet been fully elucidated. The aim of this study was to evaluate the functional and antagonistic properties of three strains of lactobacilli isolated from cocoa fermentation (*Lactobacillus fermentum *5.2,* L. plantarum* 6.2, and* L. plantarum* 7.1) against* Gardnerella vaginalis*. Our results show that the lactobacilli have potential use as probiotics, since they have high hydrophobicity and autoaggregation properties and effectively adhere to vaginal cells. Metabolites secreted into the culture medium and whole cells of the strains under study are capable of interfering with the growth of* G. vaginalis* to different degrees. The elucidation of the antagonistic mechanisms as well as their effect on human cells may be useful in the development of a product containing such microorganisms or products secreted by them.

## 1. Introduction

Probiotics are microorganisms capable of conferring health benefits to the host after correct administration. Lactic acid bacteria (LAB) are an integral part of the intestinal and genital microbiota of humans and other vertebrates [1].

Probiotic can act in different ways: (1) competitively occupying receptors on mucosal epithelial cells [2]; (2) inhibiting the adhesion of pathogens [3]; (3) producing antimicrobial substances such as bacteriocins, hydrogen peroxide, and organic acids [4, 5]; (4) inhibiting the synthesis of toxins or degrading cytotoxic compounds [6]; and (5) modulating the immune response [7, 8].

Most of the probiotics available on the market have human origin, due to the concept that (it was expected) their action would be accentuated in organisms of the same species from which the strain was primarily isolated. However, new studies have shown that microorganisms of extraintestinal origin, isolated from plants and fermented foods, exhibit promising effects in the treatment and prevention of numerous diseases [9–11].

Cocoa is the main agricultural product in southern Bahia, and Brazil is one of the world's largest producers, along with Ghana and Côte d'Ivoire in Africa [12]. The fermentation of cocoa beans is a process in which LAB plays an important role, because these microorganisms contribute in the formation of the sensory characteristics of the final product, chocolate [13, 14].

Interest in searching for new strains with probiotic potential has risen in the industrial and scientific sectors mainly due to the market demand for functional foods and therapeutics with lesser side effects and because of the numerous benefits attributed to these microorganisms [9, 15]. The role of LAB in cocoa fermentation has not been fully clarified, but the diversity of bacteria involved in this process makes this process/product a promising source for isolation of the prospecting strains for biotechnology applications [16].

Preliminary studies of our group showed that LAB isolated from this fermentative process were able to reduce intestinal inflammation induced in an experimental model of colitis in rats, decreasing the concentration of proinflammatory cytokines in serum, increasing level of IgA, and restoring tissue structure of the mucosa [10, 17].

Bacterial vaginosis is a clinical condition of disturbance of the native microbiota with decreased* Lactobacillus* counts and increased pathogenic microorganisms such as* Gardnerella vaginalis *[18]. Several LAB isolated from vaginal microenvironment exhibit inhibitory activity against vaginosis-associated pathogens, such as* Candida albicans* [19],* Neisseria gonorrhoeae, G. vaginalis* [20], and Enterobacteriaceae [4]. However, there are no investigations of the use of LAB isolated from cocoa fermentation in bacterial vaginosis models.

Thus, the aim of the present study was to evaluate* in vitro* functional and antagonistic probiotic features of three* Lactobacillus *strains isolated from the cocoa fermentation process against* G. vaginalis*.

## 2. Materials and Methods

### 2.1. Strains, Cell Lines, and Growth Conditions

Three strains of lactobacilli previously isolated and characterized by our research group [17] were used in this study:* Lactobacillus fermentum* 5.2,* Lactobacillus plantarum* 6.2, and* Lactobacillus plantarum* 7.1.

*Lactobacillus *strains were grown in de Man, Rogosa, and Sharpe (MRS) medium (HiMedia) for 18–24 h at 37°C under microaerophilic conditions.* Gardnerella vaginalis* ATCC 49154 was grown on 5% blood agar plates (HiMedia) or Brain and Heart Infusion (BHI) broth (Difco) for 18–24 h at 37°C in a 5% CO_2_ atmosphere.

HMVII, a vaginal epithelial cell line (BCRJ 0316), was grown in RPMI 1640 medium (HyClone) supplemented with 10% fetal bovine serum (FBS) (Gibco) and 1% antibiotic (penicillin and streptomycin) (Gibco) at 37°C in a 5% CO_2_ atmosphere.

### 2.2. Lactobacilli Supernatant Preparation

Lactobacilli were grown in MRS broth for 48 h at 37°C. After incubation, the supernatants were obtained by harvesting of cells by centrifugation for 15 min at 8,000 ×g. pH of supernatants was measured before lyophilization. Lyophilized supernatants were kept under refrigeration conditions until use. Before use they were reconstituted in sterile ultrapure water and filtered through 0.22 *μ*m membranes.

### 2.3. Autoaggregation and Coaggregation Assays

Autoaggregation and coaggregation assays were adapted from Kos et al. [21]. For the autoaggregation assay, strains of lactobacilli were grown in MRS broth for 18 h. After centrifugation (8,000 ×g, 10 min), pellets of cells were resuspended, washed twice with 0.9% saline, and finally resuspended to 1 × 10^8^ CFU mL^−1^ in the same solution. Then, suspensions were vortexed and incubated at 37°C for 5 h. Each hour, an aliquot (1 mL) from the top of the suspensions was carefully removed and its absorbance read at 600 nm in a spectrophotometer. Autoaggregation was calculated using the following formula: autoaggregation (%) = ((*A*_0_ − *A*_*t*_)/*A*_0_) × 100, where *A*_0_ indicates the absorbance at time 0 h and *A*_*t*_ indicates the absorbance every hour, up to 5 h.

For the coaggregation assay, a* Lactobacillus* suspension was prepared similar to the autoaggregation assay. A suspension of* G. vaginalis* cells after growth in BHI was made and finally standardized to 1 × 10^8^ CFU mL^−1^ in 0.9% saline. One ml of each* Lactobacillus* suspension was mixed with the same volume of* G. vaginalis* cell suspension and the mixture was vortexed for 10 sec and left for gravity sedimentation. Control tubes containing 2 mL of each bacterial cell suspension alone were made. Absorbance of the suspensions was read at 600 nm in a spectrophotometer after 5 h of incubation at 37°C. Coaggregation was calculated using the following formula: coaggregation (%) = [(*Ax* + *Ay*)/2 − *A*(*x* + *y*)]/[(*Ax* + *Ay*)/2], where *x* and *y* indicate the absorbance of strains in the control tubes and (*x* + *y*) indicates the absorbance of the mixtures.

### 2.4. Microbial Hydrophobicity Assay

To determine the degree of hydrophobicity, we used microbial adhesion to hydrocarbons (MATH), adapted from Rodríguez et al. [22], using xylene as solvent. Lactobacilli strains were grown in MRS broth for 18 h. After centrifugation (8,000 ×g, 10 min), pellets were recovered, washed twice with 0.9% saline, and adjusted to an optical density (OD 600) of 0.7. The solvent (xylene; 1 mL) was then added to each bacterial suspension and the mixtures were vortexed vigorously for 2 min and incubated for 2 h at 37°C. The lower aqueous phase was carefully removed and read at 600 nm in a spectrophotometer. Hydrophobicity was calculated using the following formula: hydrophobicity (%) = ((*A*_0_ − *A*_2_)/*A*_0_) × 100, where *A*_0_ indicates the absorbance at time 0 h and *A*_2_ indicates the absorbance after 2 h.

### 2.5. *Lactobacillus* Adhesion to HMVII Cells

For the adhesion test, we used a methodology adapted from Santos et al. [7]. Vaginal epithelial cells (HMVII) were used at a concentration of 1 × 10^6^ cells mL^−1^. Lactobacilli were grown in MRS broth for 18 h. After centrifugation (8,000 ×g, 10 min), pellets were recovered, washed twice with 0.9% saline, and adjusted to 1 × 10^8^ CFU mL^−1^ in RPMI supplemented with 10% FBS. Lactobacilli cell suspension was added to wells containing HMVII cells (multiplicity of infection, MOI = 100) and incubated at 37°C in a 5% CO_2_ atmosphere. Medium was added to the wells containing HMVII cells as a negative control. After 2 h of interaction, the cell monolayer was washed three times with 0.9% saline and treated with 0.25% trypsin-EDTA for 5 min. The determination of adhered lactobacilli was performed by serial dilution followed by plating on MRS agar. Plates were incubated for 48 h at 37°C and the colony forming units (CFU mL^−1^) were counted. The percentage of adhered lactobacilli was calculated by the following formula: adhesion (%) = (CFU_end_/CFU_initial_) × 100.

In addition, scanning electron microscopy (SEM) was performed to visualize lactobacilli adhered to the vaginal cells after interaction. HMVII cells (1 × 10^6^ cells mL^−1^) were grown on glass coverslips with each one of the three strains of* Lactobacillus* tested in this study (1 × 10^8^ UFC mL^−1^) and incubated for 2 h at 37°C in a 5% CO_2_ atmosphere. HMVII cells alone were used as control. Coverslips were washed three times with 0.9% saline and fixed in 2.5% glutaraldehyde in 0.1 M sodium cacodylate buffer pH 7.2. Next, dehydration was performed in series of increasing acetone concentrations (50–100%, 10 min each). The samples were subjected to critical point drying and metallized with an approximately 20 nm thick gold layer to be observed in the scanning electron microscope Quanta 250 (FEI Company).

### 2.6. Antimicrobial Activity of* Lactobacillus* Culture Supernatants

First, an evaluation of the antimicrobial activity was made through the agar diffusion technique. The strain of* G. vaginalis* was previously cultured for 18–24 h at 37°C in a 5% CO_2_ atmosphere onto blood agar plates. Afterwards, bacteria were harvested from the agar, washed with 0.9% saline, centrifuged, resuspended in the same solution, and adjusted in a spectrophotometer at the concentration of 1 × 10^8^ CFU mL^−1^. The inoculum was spread over the surface of Petri dishes containing BHI agar (Difco); then wells were perforated in the agar in which culture supernatants of the different lactobacilli were added. Plates were incubated for 24 h at 37°C in a 5% CO_2_ atmosphere. After incubation, the presence or absence of inhibition halos around the wells was observed.

Microdilution technique was performed to determine minimum inhibitory concentration (MIC) in 96-well microtiter plates according to the recommendations of the Clinical and Laboratory Standards Institute, CLSI [23]. Serial dilutions were done starting from 40 mg mL^−1^ of the culture supernatants of the lactobacilli in Mueller-Hinton broth (MH) containing 5 × 10^5^ CFU mL^−1^ of* G. vaginalis*. The same procedure was done with the following controls: lyophilized culture medium without lactobacilli (MRS control); MH without inoculum (control of sterility of the medium); MH containing 5 × 10^5^ CFU mL^−1^ of* G. vaginalis *(positive control); and MH containing 5 × 10^5^ CFU mL^−1^ of* G. vaginalis* and 12.5 *μ*g mL^−1^ of chloramphenicol (negative control). The microtiter plates were incubated for 24 h at 37°C in a 5% CO_2_ atmosphere with inhibition being observed by the absence of turbidity in the wells. To confirm whether the supernatants had a bactericidal or bacteriostatic effect, the contents of the wells were plated onto blood agar and then incubated at 37°C in a 5% CO_2_ atmosphere for 24 h [24].

### 2.7. Coculture Assay

To evaluate the influence of lactobacilli on the growth of* G. vaginalis*, we used the bacterial coculture technique described by Coudeyras et al. [25]. The assay was performed in BHI medium supplemented with 1% yeast extract, 0.1% maltose, 0.1% glucose, and 10% fetal bovine serum. An inoculum of 1 × 10^8^ CFU mL^−1^ was made for each microorganism. The strain of* G. vaginalis* was cultivated alone (control) or with each strain of the three lines of* Lactobacillus*, in a ratio of 1 : 1 at 37°C in a 5% CO_2_ atmosphere for 24 h. Aliquots were removed after 4, 8, and 24 h, serially diluted, and plated onto blood agar plates to determine the microbial count of* G. vaginalis* after interaction. Plates were also incubated at 37°C for 24 h in a 5% CO_2_ atmosphere.

### 2.8. Lactobacilli Susceptibility to Antibiotics

Susceptibility of* Lactobacillus* strains to antimicrobials was determined by the modified agar diffusion method of CLSI [26]. Lactobacilli strains were grown in MRS broth for 18 h. After centrifugation (8,000 ×g, 10 min), pellets were recovered, washed twice with 0.9% saline, and adjusted to 0.5 on the McFarland scale. One hundred microliters of this suspension was spread onto MRS agar plates, followed by the arrangement of antibiotic disks. Plates were incubated at 37°C for 18–24 h and the diameters of the halos were measured and classified as sensitive (S), moderately sensitive (MS), and resistant (R), according to Charteris et al. [27]. The antimicrobials tested were amikacin (30 *μ*g), amoxicillin (10 *μ*g), ampicillin (10 *μ*g), cefalotin (30 *μ*g), ciprofloxacin (5 *μ*g), clindamycin (2 *μ*g), chloramphenicol (30 *μ*g), erythromycin (10 *μ*g), gentamicin (10 *μ*g), nitrofurantoin (300 *μ*g), norfloxacin (10 *μ*g), penicillin G (10 *μ*g), tetracycline (30 *μ*g), and vancomycin (30 *μ*g).

### 2.9. Statistical Analysis

All experiments were performed in triplicate. Quantitative data are presented by mean and standard deviations that were analyzed using GraphPad Prism 5.01. Statistical differences between mean values were determined using ANOVA and Tukey's posttest with *p* < 0.05.

## 3. Results and Discussion

### 3.1. Autoaggregation, Hydrophobicity, and Adhesion to Vaginal Epithelial Cells

All three strains of lactobacilli tested in this study showed percentage of autoaggregation around 30% after 5 h of incubation (Table 1). Autoaggregation is an important bacterial feature in several ecological niches, especially in human and animal mucosa, where probiotics display their activities. The ability to autoaggregate (form floccules) is a crucial factor for the maintenance of significant counts of the probiotic strain in the adverse conditions present in the oral cavity and the gastrointestinal and urogenital tracts [28]. Lactobacilli, in general, have an autoaggregation capacity ranging from low to moderate [29]. In the present study, lactobacilli showed moderate autoaggregation close to or above those found for lactobacilli isolated from other fermented foods, including cocoa. Two strains of* L. plantarum* isolated from cocoa fermentation showed autoaggregation values of 18.08 and 20.94% [30]. Similarly, seven* L. fermentum* strains isolated from fermented Chinese products presented autoaggregation ranging from 0.86 to 65.15% [31]

Hydrophobicity, also known as microbial adhesion to hydrocarbons (MATH), together with autoaggregation, is considered an important bacterial surface feature and can be classified into 3 categories: low (MATH < 33%), medium (33% < 66%), or high (MATH > 66%) [29]. In this study, hydrophobicity was evaluated by the microbial adhesion to xylene (an apolar solvent) and, after 2 h of incubation, results obtained for the three strains were* L. fermentum* 5.2 and* L. plantarum *6.2 showed moderate hydrophobicity (53.96% and 55.52%, resp.) while* L. plantarum *7.1 was highly hydrophobic (71.20%). These values of hydrophobicity are much higher than those found for other lactobacilli isolated from cocoa fermentation. Ramos et al. [30], testing a strain of* L. fermentum* and three strains of* L. plantarum*, obtained hydrophobicity values ranging from zero to 1.4%. Santos and coauthors [16], analyzing hydrophobicity of 3 strains of* L. fermentum* and 6 strains of* L. plantarum*, obtained values that varied from 3.5 to 16.9%, with the highest value attributed to a strain of* L. plantarum*.

**Figure 1:**
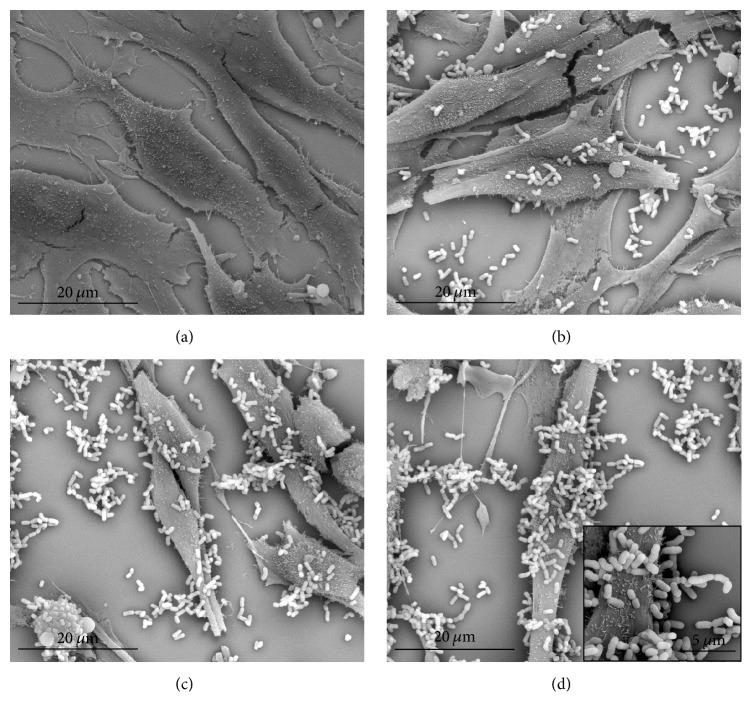
Scanning electron microscope images of vaginal epithelial cells treated for 2 h with lactobacilli isolated from cocoa fermentation. (a) Untreated HMVII cells (×2,500); (b) HMVII cells treated with* L. fermentum* 5.2 (×2,500); (c) HMVII cells treated with* L. plantarum* 6.2 (×2,500); (d) HMVII cells treated with* L. plantarum* 7.1 (×2,500; details in ×20,000).

Regarding the adhesion of* Lactobacillus* to HMVII epithelial vaginal cells, the strains* L. fermentum* 5.2 and* L. plantarum* 6.2 showed similar or almost equal percentage (35.61% and 38.78%, resp.), whereas* L. plantarum* 7.1 was significantly more adhesive (55.75%). It was possible to confirm this result by scanning electron microscopy images.* L. plantarum* 7.1 presented more bacteria adhered to HMVII cells when compared to the other two strains (Figure 1). Several studies correlate the ability of a probiotic strain to bind to host mucosal cells with autoaggregation and hydrophobicity acting synergistically [4, 29, 31]. This fact corroborates our data, where* L. plantarum* 7.1 expressed higher adhesion because it had significantly higher hydrophobicity than the other strains tested (Table 1). Studies employing lactobacilli isolated from environmental or intestinal samples showed a low adhesion to epithelial cells, usually around 10% [32, 33], a value much lower than that found with strains isolated from cocoa fermentation.

Miljkovic et al. [3] have demonstrated that extraintestinal strains of* L. paracasei* subsp.* paracasei* express AggLb, an aggregation-promoting factor that contributes to the diverse functions and behavior of the carriers, including strong aggregation and hydrophobicity abilities and strong and specific interaction with collagen through changes to cell-surface properties. AggLb is also involved in protection of the host from pathogen infection by a mechanism of competitive exclusion.

Bacterial surface properties (autoaggregation and hydrophobicity), as well as adhesion to host cells, are important criteria for the selection of probiotic bacteria strains [9, 21, 28]. Our findings show that the three tested strains have a good profile that could be used as vaginal probiotics.

### 3.2. Anti-*Gardnerella *Activity

**Figure 2:**
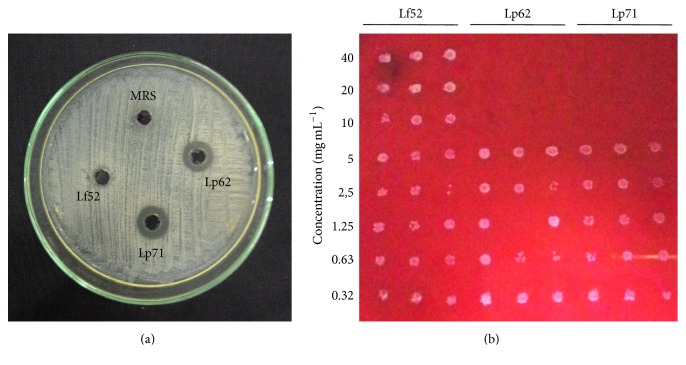
Evaluation of antimicrobial activity of culture supernatants of lactobacilli isolated from cocoa fermentation against* Gardnerella vaginalis*. (a) Evaluation by agar diffusion. (b) Determination of minimum inhibitory concentrations. MRS: culture medium; Lf52:* L. fermentum* 5.2; Lp62:* L. plantarum* 6.2; and Lp71:* L. plantarum* 7.1.

Using the agar diffusion technique, we observed that only the supernatants of the* L. plantarum* strains used in this study (but not* L. fermentum *5.2) showed antimicrobial activity against* G. vaginalis*, notable by the presence of inhibition halos around the wells. The supernatant halos of* L. plantarum* 6.2 and* L. plantarum* 7.1 were 12 and 11 mm, respectively (Figure 2(a)).

Also in microdilution test where the minimum inhibitory concentration (MIC) was determined, inhibition of* G. vaginalis* was observed after exposure to the supernatants of both* L. plantarum* strains but not to* L. fermentum*. The minimum inhibitory concentration of both* L. plantarum* 6.2 and* L. plantarum* 7.1 supernatants was 10 mg mL^−1^. This effect was considered as bactericidal and confirmed by plating the contents of each well on the plate. After 24 h of incubation, there was no bacterial growth when* G. vaginalis *culture was exposed to 10 mg mL^−1^ or higher concentrations of culture supernatants (Figure 2(b)).

Antibacterial activity of* L. plantarum* supernatants alone may be related to their acidity, since the supernatants of* L. plantarum* 6.2 and* L. plantarum* 7.1 had pH of 3.81 and 3.77, respectively, while the pH of the supernatant of* L. fermentum* 5.2 was 4.78 (Table 1). The culture medium without any microbial growth had pH of 6.61. Some studies report that the difference in acid production is species-dependent in lactobacilli isolated from diverse sources. Supernatant of* L. plantarum* strain WSO, isolated from cucumber fermentation, had pH of 3.81 [34]. On the other hand, supernatant of a vaginal isolated* L. fermentum* with inhibitory potential against* G. vaginalis* had a pH of 4.16 [35]. Poppi et al. [36] showed that the pH of the supernatants of two* L. plantarum* strains (22c and 41b) isolated from poultry litter was 3.83 and 3.88, respectively.

Studies by other authors using the agar diffusion technique have shown that lactobacilli culture supernatants isolated from the vaginal microenvironment displayed inhibitory activity against* Escherichia coli*,* Staphylococcus aureus*,* Proteus vulgaris*,* Klebsiella pneumoniae*, and* Gardnerella vaginalis *[37–40]. After adjustment of pH to 6.5, the number of inhibitory strains was reduced to less than half of that observed when the supernatant was used without any treatment, indicating an important role of acids derived from the metabolism of lactobacilli in antibacterial activity [37]. The same effect was observed by Onwuakor et al. [38], where maize-isolated lactobacilli culture supernatants lost inhibitory activity against* Salmonella typhimurium* and* Shigella dysenteriae* when the pH was adjusted to values above 7.0. Antagonistic effects related to acid production (mainly lactic acid) have already been demonstrated for lactobacilli isolated from several sources. In these studies, exposure to high temperature or protease treatments did not significantly alter the antimicrobial activity of culture supernatants [39, 40].

In a study conducted by Melo et al. [24], the culture supernatant of an* L. fermentum* strain isolated from cocoa fermentation was able to inhibit the growth of* S. aureus* with an MIC of 20 mg mL^−1^. This effect, as found in our study for* L. plantarum *strains, was bactericidal and was confirmed by plating of treated culture. Similarly, a culture supernatant of a* L. paracasei* strain isolated from fermented milk was also shown to inhibit bacterial growth of pathogens, especially* E. coli*, with an MIC of 15.6 mg mL^−1^ [41]. However, to achieve the same effect on* Serratia marcescens*, values around 0.16 mg mL^−1^ of the culture supernatants from strains belonging to the species* L. acidophilus* and* L. plantarum* were required [5]. The activity of the culture supernatants against pathogens depends on several factors that include (1) the susceptibility of the target microorganism and (2) the composition of the lactobacilli supernatants, which differs in relation to the species, strain, and source of isolation, justifying the variation of MICs found in different studies [42].

The three strains of lactobacilli tested in our study showed high coaggregation values after incubation with* G. vaginalis*, greater than 40% (Table 1). Reduction of the adhesive activity of* G. vaginalis* bacteria by* Lactobacillus* strains is a well-known and desired effect of strains for potential vaginal probiotic application. In fact, other authors found that vaginal isolates of* L. acidophilus*,* L. gasseri*, and* L. jensenii* showed high coaggregation activity against* C. albicans*,* E. coli*, and* G. vaginalis* [4]. Mastromarino et al. [43] demonstrated high efficiency of coaggregation of* L. salivarius* and* L. gasseri* with* G. vaginalis*. In addition, strains of* L. fermentum *and* L. plantarum* isolated from cocoa fermentation efficiently coaggregated with* E. coli*,* Shigella flexneri*,* Salmonella enterica*,* L. monocytogenes*, and* S. aureus* [16, 30]. Coaggregation of probiotic microorganisms to pathogens generates a hostile environment for the pathogens implying the reduction of their growth, facilitation of the removal of the pathogen, and reestablishment of indigenous microbiota [44].

**Figure 3:**
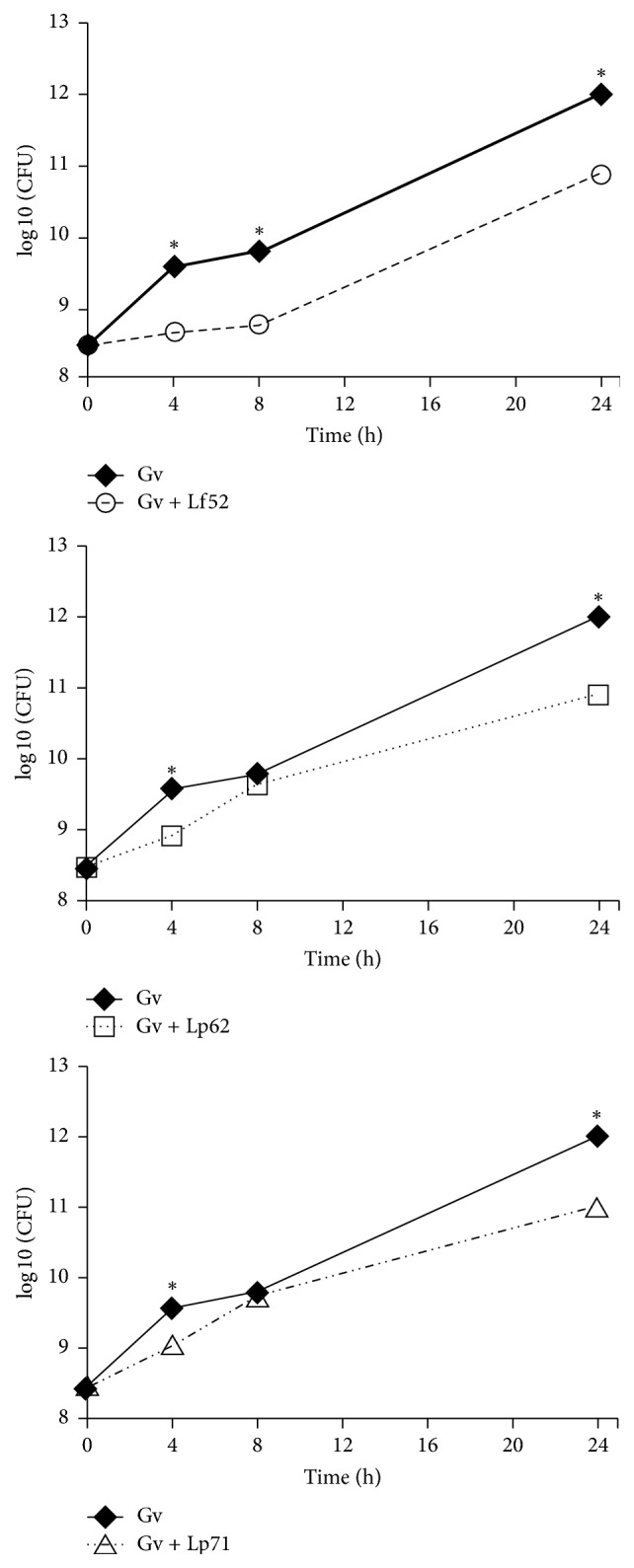
Effect of lactobacilli isolated from cocoa fermentation on the viability of* Gardnerella vaginalis *(Gv) as a function of the time of coculture. The pathogen was incubated without (filled shape) or with (empty shape) different lactobacilli (*L. fermentum *5.2: Lf52;* L. plantarum *6.2: Lp62; or* L. plantarum *7.1: Lp71) for 24 hours and CFU mL^−1^ was determined after 4, 8, and 24 hours of incubation by plating onto appropriate media. Each value shown is the mean ± SD. ^*∗*^Statistically significant differences (*p* < 0.05).

The coculture technique is able to assess the influence of one microorganism on the growth of another when both are incubated together. We observed that all* Lactobacillus* strains were able to reduce by one log unit the microbial counts of* G. vaginalis* after 24 hours of incubation when compared to* G. vaginalis* growing alone (Figure 3). Only* L. fermentum* 5.2 was able to maintain inhibitory activity against* G. vaginalis* during all the time period evaluated. It has been previously found that* L. acidophilus*,* L. jensenii*,* L. gasseri*, and* L. crispatus* isolated from the vaginal microbiota of healthy women showed inhibitory activity, demonstrated by the coculture technique, against* G. vaginalis* and* Prevotella bivia* with stable inhibition from the first hour [45]. These results were similar to those found by Coudeyras et al. [25] who, using a* L. rhamnosus* strain, demonstrated inhibition of* G. vaginalis*,* P. bivia*, and* C. albicans* after 8 hours, with significant inhibition of* G. vaginalis* after 24 hours. Other pathogens that are also capable of causing bacterial vaginosis, such as* E. coli* and* S. aureus*, also have their growth affected when cocultivated with strains of* L. plantarum* and* L. fermentum*: after 24 hours, a decrease of up to three logs was observed when compared to controls [46].

In the present study, a concentration of 10^8^ lactobacilli per mL was used in the coaggregation and coculture assays. Results found were satisfactory and promising, since such concentration was able to inhibit the growth of* G. vaginalis* after interaction. Commercial formulations and* in vivo* studies show that a concentration ranging from 10^8^ to 10^9^ CFU is required to achieve the same result [47–49].

### 3.3. Antimicrobial Susceptibility

Susceptibility of lactobacilli isolated from cocoa fermentation to different antimicrobials is shown in Table 2. Although lactobacilli have a long history of safe use, under certain host conditions they may cause rare bacteremia and endocarditis. Thus, some safety tests should be performed, such as antimicrobial susceptibility [4, 50]. The three strains of lactobacilli were sensitive to most antimicrobials tested and resistant to following antibiotics: vancomycin (a glycopeptide), aminoglycosides, and quinolones. Lactobacilli are generally resistant to antimicrobial inhibitors of nucleic acid synthesis, such as quinolones, whereas they are sensitive to cell wall inhibitors and protein synthesis inhibitors, except for vancomycin and aminoglycosides, respectively. It is important to emphasize that resistance to such antimicrobials is intrinsic to the genus* Lactobacillus* and does not present a risk of being transferred through horizontal genetic transfer to the bacteria of the native intestinal microbiota [9, 16, 51, 52].

## 4. Conclusion

Lactobacilli used in this study may protect the vaginal environment through multiple mechanisms, including adhesion to the epithelium, coaggregation with potential pathogens, and production of antagonistic molecules. They are promissory strains for the development of prophylactic agents. These results may serve as a basis for further studies aimed at investigating molecular mechanisms related to the inhibition of* G. vaginalis* by lactobacilli and their metabolites, as well as evaluating the immunomodulatory capacity of lactobacilli isolated from cocoa fermentation.

## Figures and Tables

**Table 1:** Characterization of surface properties, adhesion to vaginal cells, and antimicrobial properties of lactobacilli isolated from cocoa fermentation.

Strain	Surface properties		Adhesion to HMVII cells (%)	Antimicrobial properties	
	Autoaggregation (%)	Hydrophobicity (%)		Coaggregation with *G. vaginalis *(%)	Acidification
*L. fermentum* 5.2	31.18 ± 4.39^a^	53.96 ± 2.90^a^	35.61 ± 2.98^a^	43.15 ± 0.68^a^	4.78
*L. plantarum* 6.2	33.44 ± 1.53^a^	55.52 ± 3.76^a^	38.73 ± 2.87^a^	44.61 ± 0.17^a^	3.81
*L. plantarum* 7.1	29.23 ± 1.14^a^	71.20 ± 3.03^b^	55.75 ± 3.72^b^	44.15 ± 0.51^a^	3.77

Presented values are means of triplicate determinations; ± indicates standard deviations from the mean. Mean values (±standard deviation) within the same column followed by different superscript letters differ significantly (*p* < 0.05).

**Table 2:** Susceptibility profile of *Lactobacillus* strains isolated from cocoa fermentation.

Antimicrobial			Susceptibility^a^		
Group	Name	Disc conc. (*µ*g)	*L. fermentum* 5.2	*L. plantarum *6.2	*L. plantarum* 7.1
*Inhibitors of cell wall synthesis*					
Penicillin	Amoxicillin	10	S	S	S
	Ampicillin	10	S	S	S
	Penicillin G	10	S	MS	MS
Cephalosporins	Cefalotin	30	S	S	S
Glycopeptides	Vancomycin	30	R	R	R
*Inhibitors of protein synthesis*					
Aminoglycosides	Amikacin	30	R	R	R
	Gentamicin	10	R	R	R
	Streptomycin	10	R	R	R
Tetracyclines	Tetracycline	30	S	S	MS
Single antibiotics	Chloramphenicol	30	S	S	S
Macrolides	Erythromycin	15	S	S	S
Lincosamides	Clindamycin	2	S	S	S
*Inhibitors of nucleic acid synthesis*					
Quinolones	Ciprofloxacin	5	R	R	R
	Norfloxacin	10	R	R	R
*Other urinary tract antiseptics*					
Single antibiotics	Nitrofurantoin	300	S	S	S

^a^Susceptibility expressed as sensitive (S), moderately sensitive (MS), or resistant (R) [18].
